# Hospital Saturday Fund

**Published:** 1902-01-04

**Authors:** 


### HOSPITAL SATURDAY FUND.

A meeting of the committee of the Metropolitan Hospital Saturday Fund was held on December 27th, when it was reported that the receipts from the workshops, etc., from January 8th to December 7th had reached £15,693 19s. 7d., as compared with £15,060 at the corresponding period of last year. The expenditure since January 8th was £1,906 3s. 2d. The sum of £10,500 had been placed on deposit at the bankers and £3,134 16s. 1d. advanced to the premises fund. The board of delegates, in their quarterly official "circular" just issued, state that "there is one very important difference between the Prince of Wales's Fund and the Hospital Saturday Fund from the subscribers' point of view. The contributions to the former must be considered a free gift to the hospitals and no 'letters' will be given in exchange. To depart from this leading principle of the fund would defeat the object for which it was established. On the other hand, the Hospital Saturday Fund has now become much more than a collecting agency. During each year the committee of the fund distributes over 26,000 letters of recommendation for the various institutions, including letters for the supply of surgical appliances and for sending men, women, and children to suitable convalescent homes. A special department has also been created through which men and women are trained to render first aid in cases of accidents. There is no doubt these advantages are highly valued by the subscribers, as they are, unfortunately, a necessity of their condition of life and employment. We hope that the present year will result in a substantial increase in the sum collected by the fund, and we have every indication that the fund is growing in popularity among the workmen of London. We sincerely hope that the time is not far distant when the three organisations—viz. the Prince of Wales's Fund, the Hospital Sunday Fund, and the Hospital Saturday Fund—will become one great organisation with three branches, each working in its own department. At the end of each year the proceeds could well be distributed by one distribution committee upon principles which could be easily defined after the experience of past years. We believe that such a movement as this would secure the confidence of the public and do more to relieve the institutions of their present embarrassment than any other steps that could be taken."

